# Strengthening access to cancer medicines for children in East Africa: policy options to enhance medicine procurement, forecasting, and regulations

**DOI:** 10.1186/s41256-024-00365-y

**Published:** 2024-07-01

**Authors:** Kadia Petricca, Laura Carson, Joyce Kambugu, Avram Denburg

**Affiliations:** 1 Peter Gilgan Centre for Research and Learning, The Hospital for Sick Children, Toronto, ON Canada; 2 Department of Paediatric Oncology, Uganda Cancer Institute, Kampala, Uganda; 3 Department of Paediatrics, University of Toronto, Toronto, ON Canada; 4 Division of Haematology/Oncology, The Hospital for Sick Children, Toronto, ON M5G 1X8 Canada

**Keywords:** Access to medicines, Childhood cancer, Pooled procurement, Regional harmonization, Policy brief

## Abstract

Gaps in access to quality essential medicines remain a major impediment to the effective care of children with cancer in low-and middle-income countries (LMICs). The World Health Organization reports that less than 30% of LMICs have consistent availability of childhood cancer medicines, compared to over 95% in high-income countries. Information provided within this policy brief is drawn from a review of the literature and a mixed-methods study published in the Lancet Oncology that analyzed determinants of cancer medicine access for children in Kenya, Tanzania, Uganda, and Rwanda. Three key policy options are presented to guide strategic policy direction and critical health system planning for strengthening access to cancer medicines for children: pooled procurement, evidence-based forecasting, and regional harmonization of regulatory processes. Enhancing regional pooled procurement to address fragmented markets and improve medicine supply, investing in health information systems for improved forecasting and planning of childhood cancer medicine needs, and promoting regulatory harmonization to streamline medicine approval and quality assurance across East Africa are recommended. This policy brief is intended for policymakers, clinicians, and health-system planners involved in the procurement, supply chain management, policy and financing of childhood cancer medicines.

## Background

Gaps in access to quality essential medicines remain a major impediment to the effective care of children with cancer in low- and middle-income countries (LMICs) [1]. The World Health Organization (WHO) reports that less than 30% of LMICs have consistent availability of childhood cancer medicines, compared to over 95% in high-income countries (HICs) [2]. This disparity is due to a number of factors, including market inefficiencies that limit the availability of affordable products, supply chain disruptions, inadequate data for evidence-based forecasting and procurement, and limited targeted policy and financing for childhood cancer.

Childhood cancer presents significant acute and long-term challenges for patients, families, communities, and health systems. In recent decades, significant strides have been made in childhood cancer research and treatment. However, survival rates remain disproportionately low in many LMICs [2]. The 2023 WHO report, Access to NCD Medicines [3], emphasizes the marked inequities faced by patients in LMICs seeking cancer treatment. The provision of effective treatment for childhood cancer remains a significant predictor of survival and relies crucially on the sustained provision of quality chemotherapeutic and supportive care medicines. Adequate policy and financing, along with efficient procurement and supply management practices, are integral to anticipating and meeting patient-level demands and ensuring sound health system resource stewardship.

This policy brief presents three policy options to guide critical health system planning for strengthening access to cancer medicines for children: (I) pooled procurement, (II) evidence-based forecasting, and (III) regional harmonization of regulatory processes. These options emerge from a literature review and the ACCESS East Africa (EA) study that analyzed the determinants of cancer medicine access for children in Kenya, Tanzania, Uganda, and Rwanda published in the Lancet Oncology in May 2023 [4].

## Policy option 1: investment in regional pooled procurement

**Fig. 1:**
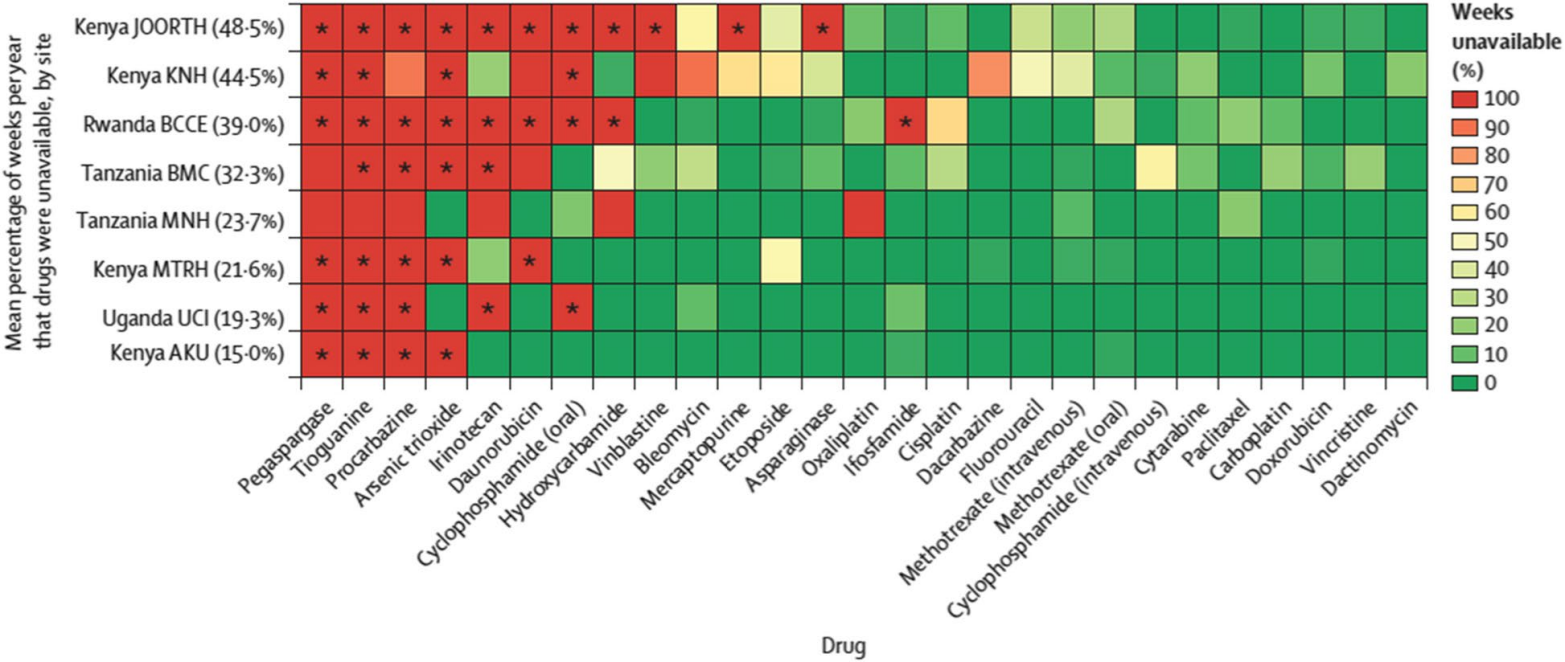
Availability of cytotoxic medicines by site. AKU: Aga Khan University Hospital. BCCE = Butaro Cancer Centre of Excellence. BMC = Bugando Medical Centre. JOORTH = Jaramogi Oginga Odinga Referral and Teaching Hospital. KNH = Kenyatta National Hospital. MNH = Muhimbili National Hospital. MTRH = Moi University Teaching and Referral Hospital. UCI = Uganda Cancer Institute. *Drug not stocked

**Fig. 2:**
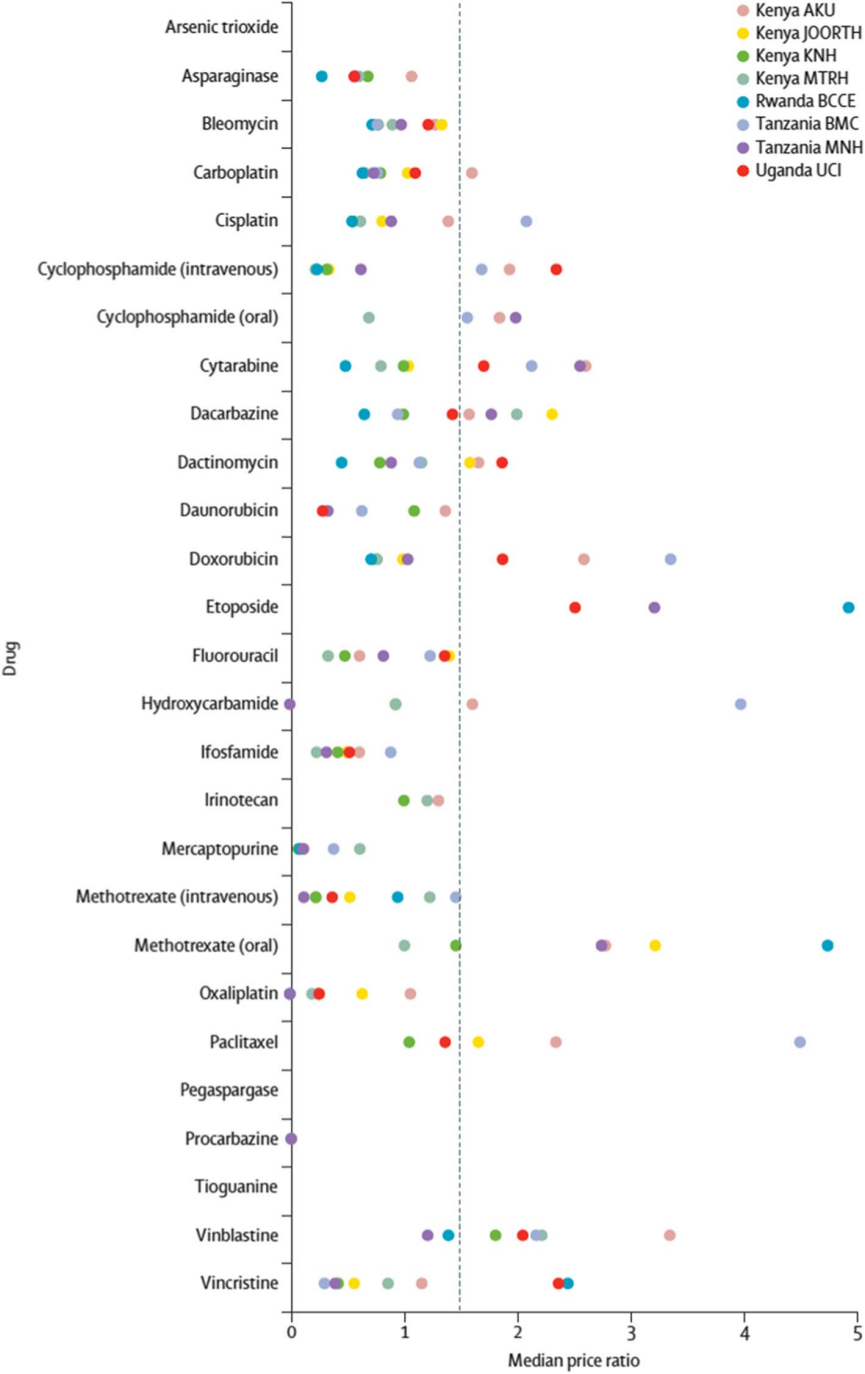
Median price ratio of each cytotoxic drug by site. The dashed line represents the accepted threshold for efficient procurement by WHO standard (median price ratio ≤ 1.5). No datapoints are provided for arsenic trioxide, tioguanine, or pegaspargase as they were not available at any point during the study period and so no data were available to calculate median price ratio. AKU: Aga Khan University Hospital. BCCE = Butaro Cancer Centre of Excellence. BMC = Bugando Medical Centre. JOORTH = Jaramogi Oginga Odinga Referral and Teaching Hospital. KNH = Kenyatta National Hospital. MNH = Muhimbili National Hospital. MTRH = Moi University Teaching and Referral Hospital. UCI = Uganda Cancer Institute

The ACCESS EA study revealed variable patterns of cytotoxic and supportive care medicine availability between May 1, 2020, and Jan 31, 2022. Stockouts were evident at both urban and rural sites, academic and community hospitals, and public and private institutions. The mean proportion of cytotoxic medicines that were unavailable across facilities ranged from 15% (lowest mean stockouts) at Aga Khan University Hospital (Kenya) to 48.5% (highest mean stockouts) at Jaramogi Oginga Odinga Hospital (Kenya) (Fig. 1) [4]. Specific medicines—including cyclophosphamide (oral and intravenous), cytarabine, dacarbazine, doxorubicin, methotrexate (oral), paclitaxel, and vinblastine—appeared more susceptible to price variation and inefficient procurement, defined as a median price ratio < 1.5 when comparing domestic and international reference prices, per WHO standards (Fig. 2) [4]. In Uganda, although procurement for cancer and supportive care medicines are institutionally centralized, the presence of selecting inefficiently procured medicines—such as vinblastine, vincristine, cyclophosphamide, etoposide, and doxorubicin—reveal opportunities for further price reductions via regional procurement mechanisms.

These findings underscore the presence of prominent but remediable procurement challenges in the East African region. These challenges principally relate to fractured, uncoordinated, and demand-insensitive approaches to procurement, premised on weak data and institutionally isolated strategies. The result is small, fragmented, and unpredictable markets for childhood cancer medicines, resulting in weak manufacturer incentives for market entry, barriers to sustained supply, and erratic inventory. To overcome these barriers, this policy option focuses on strategies to support efficient and sustained procurement of childhood cancer medicines through regional pooled procurement.

Pooled procurement is a component of supply chain management that is guided by collaboration among buyers, which motivates competition between vendors. Efforts to strengthen centralized procurement regionally can serve as an opportunity for countries to increase their bargaining power, thus reducing costs, and achieving greater predictability in their supply of essential medicines. The ACCESS EA study highlighted the significant role that pooled procurement could play in helping to secure the availability of cancer medicines for children. To guide regional pooled procurement, it is recommended that:

i. Strong political will guide alignment of shared national/regional values around transparency and data sharing to enable productive collaboration [5];
ii. Governments align policy reform for tendering and negotiation with reform for centralization of medicine tracking and regional pooled procurement [5];
iii. Sustainable and predictable financing investment is committed for a capitalization fund to support efforts for pooled procurement [6].
iv. Investment in human resource technical capacity is prioritized at the level of the buyer and pooled procurement organizing body [6].
v. Sufficient supply-side competition is pursued via the presence of multiple buyers willing to participate in the tender.

## Policy option 2: investment in health system information systems to support forecasting of childhood cancer medicines

The ACCESS EA study highlighted the significant role that historical consumption patterns have played in childhood cancer medicine planning. The need for ongoing improvements in data management systems was emphasized, coupled with health system efforts to strengthen national cancer registries. To further support efficient and sustained procurement of childhood cancer medicines, further investment is recommended to support the acquisition of quality data to guide forecasting and decision-making. Forecasting is the process of utilizing past and present data to model future demand and is critical for predicting childhood cancer care needs. Evidence-based forecasting enables health systems to adequately prepare for the anticipated needs of the population and engage in more strategic fiscal planning. High-quality and functional health information systems play an essential role in the conduct of accurate and complex forecasting. To guide improvements in forecasting, it is recommended that:

i. Policy and budgetary investments are made to strengthen national data collection on drug consumption patterns. This includes strengthening health information systems and increasing the regional cadre of pharmacists trained in inventory management and drug consumption tracking.
ii. Policy and financing investment in data management systems to support childhood cancer surveillance is strengthened.
iii. Forecasting tools are integrated into national planning for childhood cancer medicines. For example, FORxECAST [7] can estimate drug quantity and cost for 18 pediatric cancers and is readily adaptable to local health system realities.

## Policy option 3: continued investment in regulatory harmonization

The findings from the ACCESS EA study reveal the presence of regulatory barriers that impede efficient procurement and monitoring of medicine quality and pharmacovigilance across countries in the region. To overcome these barriers, this policy option focuses on recommendations to support greater investment in national regulatory reform and in existing regional regulatory harmonization efforts. The regulation of medicines seeks to ensure that patients have access to safe and efficacious medicines of high quality. Regulatory harmonization can be understood as a process in which regulatory authorities align technical requirements for the development and marketing of pharmaceutical products. This process has numerous benefits, including optimizing the use of limited resources, promoting the transfer of data, and strengthening quality assurance. When countries harmonize their approach to regulatory approval of new medicines, it unlocks the potential for pooled procurement of licensed medical products and reduces local redundancies in regulatory capacity.

In the case of childhood cancer, recent years have seen a rise in innovative therapies with both adult and pediatric indications, resulting in mounting requirements for licensing and labelling novel cancer agents in regulatory environments internationally. Harmonization of regulatory standards and processes can foster more efficient regulation of these new medicines and promote more widespread availability, particularly for LMICs. The African Medicines Regulatory Harmonization (AMRH) initiative and the East African Community Medicines Regulatory Harmonization (EAC-MRH) program are two entities that seek to support the harmonization of regulatory processes in East Africa [8]. Designing a harmonization program that is locally driven and informed will be a more suitable avenue to pursue for LMICs, including the East African region. Importantly, the WHO has both a medicines pre-qualification program (PQP) and a pediatric regulatory network, which together provide infrastructure for the streamlined review and exchange of regulatory information on pediatric medical products.

The ACCESS EA study highlighted barriers in access related to existing regulatory procedures, including significant concern regarding the safety and quality of childhood cancer medicines. A lack of adequate technology for drug quality testing was noted in addition to limited quality assurance procedures for private vendors. Furthermore, the implementation of pharmacovigilance and drug quality assessment requirements emerged as varied and requiring greater attention. These challenges indicate an urgent need for improvements in regulatory procedures, which may be facilitated through the implementation of regional strategies to harmonize standards and/or processes to strengthen opportunities for quality assurance, transparency, and accountability. Such investment would support greater confidence in the safety and quality of childhood cancer medicines. To support regional harmonization of pharmaceutical regulations, it is recommended that:

i. The WHO PQP is leveraged to streamline regional approvals in the East African Community.
ii. Resources from the WHO’s Pediatric Regulatory Network (PRN) are employed to support the harmonization of regulatory policy in East Africa [9].
iii. International Standards Organization (ISO) certification by the National Medical Regulatory Authorities (NMRAs) is pursued, where applicable, to improve regulatory efficiency.
iv. Technical and budgetary investment is made to support the AMRH initiative and the EAC-MRH program. Consider the establishment of a pilot in cancer medicines regulatory harmonization through these entities [10].
v. Regulatory reform prioritizes national pricing control strategies.

## Way forward

Access to childhood cancer medicines across East Africa is marked by gaps in availability that have implications for effective treatment delivery for a range of childhood cancers. Findings from the ACCESS EA study reveal policy windows of opportunity to support medicine access via a strategic focus on pooled procurement, evidence-based forecasting, and regional harmonization of regulatory processes. This policy brief provides a series of recommendations to guide further research as well as national and regional decision-making on optimal strategies to strengthen access to cancer medicines.

## Data Availability

The data presented herein is part of a wider collaborative study that was recently published: Petricca K, Kambugu J, Githang’a J, Macharia WM, Njuguna F, McLigeyo A, Nyangasi M, Orem J, Kanyamuhunga A, Laiti R, Katabalo D, Schroeder K, Rogo K, Maguire B, Wambui L, Nkurunziza JN, Wong B, Neposlan J, Kilawe L, Gupta S, Denburg AE. Access to essential cancer medicines for children: a comparative mixed-methods analysis of availability, price, and health-system determinants in east Africa. Lancet Oncol. (2023). S1470-2045(23)00072–4. 10.1016/S1470-2045(23)00072-4. Data relevant to the quantitative component are available upon reasonable request. To protect participant confidentiality, the qualitative data that support the findings of this study are available upon reasonable request to the corresponding author.

## Abbreviations

ACCESS EA: ACCESS East Africa
AMRH: African Medicines Regulatory Harmonization
EAC: East African Community
EAC-MRH: East African Community Medicines Regulatory Harmonization
ISO: International Standards Organization
LMIC: Low- and Middle-Income Country
GICC: Global Initiative for Childhood Cancer
NCD: Non-Communicable Disease
NMRA: National Medical Regulatory Authorities
PRN: Pediatric Regulatory Network
PQP: Prequalification Program
UHC: Universal Health Coverage
WHO: World Health Organization
